# Supplementary material for: Content-rich biological network constructed by mining PubMed abstracts
Source: BMC Bioinformatics. 2004 Oct 8;5:147. doi: 10.1186/1471-2105-5-147 (PMC528731; doi:10.1186/1471-2105-5-147)
Supplement: Additional File 5 — The original Chilibot query results of the term "long-term potentiation (LTP)" and 22 other terms, limiting the latest references analyzed to the years 1990, 1995, 2000, and 2004. [file 1471-2105-5-147-S5.bz2 › chilibotAdditionalFile5/ltp2004/html/ERK_PKC.html]

 


 **ERK** and **PKC** 
  
Found 585 abstracts in PubMed,  **30 abstracts were retrieved and analyzed**.  


---

 Search Google  |
 PDF files only 
|  EDU domain only 

---

**Interactive relationship** (e.g. stimulation, inhibition, etc)

- However, TCR induced  **ERK**  activation was suppressed by inhibitors of  **PKC**  and PLC gamma.  Ref: 14680820 Biochem Biophys Res Commun, 2003
- These results taken together suggest that lysophospholipid receptor activation involves receptor coupling to heterotrimeric Gq subunits with consequent activation of PLC,  **PKC** , and MAPK pathways leading to  **ERK**  phosphorylation.  Ref: 14648542 Glia, 2004
- **ERK**  activation by GnRH is dependent on protein kinase C  **PKC** , as evident by activation, inhibition and depletion of TPA sensitive  **PKC**  subspecies.  Ref: 14736735 EndocrinologyEndocrinology, 2004
- Our results suggest a critical involvement of  **PKC**  and tyrosine phosphorylation in OxPAPC induced activation of  **Erk**  MAP kinase cascade associated with regulation of specific gene expression, and demonstrate rapid phosphorylation of cytoskeletal proteins, which indicates OxPAPC induced EC remodeling.  Ref: 14709399 Microvasc Res, 2004
- Thus, PLD2 could be involved in MEK  **ERK**  signaling cascades that are induced by the VEGF VEGFR 2  **PKC**  delta pathway in endothelial cells.  Ref: 14704231 Am J Physiol Heart Circ PhysiolAm J Physiol Heart Circ Physiol, 2004
- **ERK**  activation was found to be required for critical downstream effects of  **PKC**   **PKC**  a activation, including cyclin D1 downregulation, p21waf1 cip1 induction, and cell cycle arrest.  Ref: 14670956 J Biol ChemJ Biol Chem, 2004
- Taken together our results indicated that the kinetic profile of  **ERK**  activation and its impact on cell proliferation can be modulated by the differential involvement of  **PKC**  isoforms and the amplitude of Ras activation.  Ref: 14644778 Am J Physiol Cell PhysiolAm J Physiol Cell Physiol, 2004
- Furthermore, analysis of the signaling events linking  **PKC**   **PKC**  a activation to changes in the cell cycle regulatory machinery implicate the Ras Raf MEK  **ERK**  cascade.  Ref: 14670956 J Biol ChemJ Biol Chem, 2004
- **PKC**  inhibition also impaired context and tone dependent fear conditioning and prevented the phosphorylation of  **Erk**  1 2, Elk 1, and CREB, without affecting p90Rsk 1.  Ref: 14739006 Behav Brain Res, 2004
- **PKC**   **PKC**  a activity promoted GTP loading of Ras, activation of Raf 1, and phosphorylation activation of  **ERK** .  Ref: 14670956 J Biol ChemJ Biol Chem, 2004
- LPA and S1P induced  **ERK**  phosphorylation was dependent on the activation of mitogen activated protein kinase MAPK, phospholipase C PLC, and protein kinase C  **PKC** , but was insensitive to pertussis toxin PTX.  Ref: 14648542 Glia, 2004
- Inhibition and down regulation of  **PKC**  demonstrated that  **ERK**  activation at 5 min involved  **PKC**  delta and zeta for ET 1, and  **PKC**  alpha plus another  **PKC**  isoform for PDGF.  Ref: 14644778 Am J Physiol Cell PhysiolAm J Physiol Cell Physiol, 2004
- Assays with inhibitors and antibodies against proteins involved in signal transduction demonstrated that NAPS and TAPS elevated COX 2 expression via tyrosine kinase, src, PI 3 kinase and  **PKC** , followed by  **ERK**  activation.  Ref: 14708615 J Invest Dermatol, 2003
- Protein kinase C  **PKC**  and phosphatidylinositol 3 kinase PI 3 K are possible alternative kinases involved in the  **ERK**  signaling pathway.  Ref: 14705145 J Neurosci Res, 2004
- Comparison of the effects of  **PKC**  agonists that differ in their ability to sustain  **PKC**  a activation and growth arrest in IEC 18 cells, together with the use of selective kinase inhibitors, indicated that the length of  **PKC**  mediated cell cycle exit is dictated by the magnitude duration of input signali.e.,  **PKC**  a activity and of activation of the  **ERK**  cascade.  Ref: 14670956 J Biol ChemJ Biol Chem, 2004
- Pharmacological inhibition of  **PKC**  and tyrosine kinases blocked activation of  **Erk**  kinase cascade upstream of Raf.  Ref: 14709399 Microvasc Res, 2004
- In the present studies the neuroprotective activity of rasagiline is blocked by  **ERK**  inhibitor, PD98059 20 microM, suggesting the involvement of  **PKC**  MAP kinase pathway in the neuroprotection.  Ref: 14732458 Neurosci Lett, 2004

**Parallel relationship** (e.g. studied together, co-existance, homology, etc.)

- **PKC**  signaling promoted cytoplasmic and nuclear accumulation of  **ERK**  activity, while growth factor induced phospho  **ERK**  was localized only in the cytoplasm.  Ref: 14670956 J Biol ChemJ Biol Chem, 2004
- Receptor and kinase activation is linked to distal signaling pathways PLC gamma1 activation, calcium influx,  **PKC**  activation and Ras  **Erk**  activation via the adapter protein LAT Linker for Activation of T cells.  Ref: 14696041 Bioessays, 2004
- Retinoic acid induced neuritogenesis of human neuroblastoma SH SY5Y cells is  **ERK**  independent and  **PKC**  dependent.  Ref: 14705145 J Neurosci Res, 2004
- These findings suggest that intracellular tyrosine kinases, MEK  **ERK**  and PI3 kinase AKT pathways are involved in 5 S HETE stimulated pancreatic cancer cell proliferation but P38 kinase, JNK SAPK and  **PKC**  are not involved in this mitogenic effect.  Ref: 14707447 Oncology, 2003
- In fact, in the presence of the specific  **PKC**  inhibitor GF 109203X, or the specific PI 3 K inhibitor wortmannin, we observed a significant dose dependent reduction in  **ERK**  phosphorylation.  Ref: 14705145 J Neurosci Res, 2004
- These results indicated that PMA up regulates SPHK1 gene expression through PMA responsive elements of the 5 promoter area of the gene, and suggested that PMA mediated SPHK1 gene expression would be mediated via  **PKC**  and  **ERK**  dependent signal transduction pathway by binding the transcription factor to AP 2 motifs.  Ref: 14729073 Biochim Biophys Acta, 2003
- We investigated the involvement of hippocampal protein kinase A PKA, protein kinase C  **PKC** , calcium calmodulin dependent protein kinases CaMK II, and mitogen activated extracellular signal regulated kinases 1 and 2 Mek 1 2 in the phosphorylation of their downstream targets extracellular signal regulated kinases 1 and 2  **Erk**  1 2, cAMP response element binding protein CREB, Elk 1, and p90 ribosomal S6 kinase 1 p90Rsk 1.  Ref: 14739006 Behav Brain Res, 2004
- On the basis of these approaches, the cyclic AMP protein kinase PKA, protein kinase C  **PKC**  and extracellularly regulated protein kinases 1 and 2  **Erk**  1 2 have been identified as the core signaling pathways in memory consolidation.  Ref: 14673206 NeurosignalsNeurosignals, 2004
- **PKC**  induced  **ERK**  activation was strong and sustained relative to that produced by proliferative signals, and the growth inhibitory effects of  **PKC**  agonists were dominant over proliferative events when these opposing stimuli were administered simultaneously.  Ref: 14670956 J Biol ChemJ Biol Chem, 2004
- Taken together, the data point to  **PKC**  a and the Ras Raf MEK  **ERK**  cascade as key regulators of cell cycle withdrawal in intestinal epithelial cells.  Ref: 14670956 J Biol ChemJ Biol Chem, 2004
- Epidermal growth factor also inhibited CD47 induced apoptosis via a  **PKC**  dependent but  **ERK**  independent pathway.  Ref: 14871834 Cancer Res, 2004
- Thus,  **PKC** ,  **ERK** , JNK, p38 and c Src... are involved in GnRH induction of the oFSHbeta gene.  Ref: 14736735 EndocrinologyEndocrinology, 2004
- Our results demonstrate that OxPAPC treatment activated in a time dependent fashion protein kinase C  **PKC** , protein kinase A PKA, Raf MEK  **Erk**  MAP kinase cascade, JNK MAP kinase and transient protein tyrosine phosphorylation in human pulmonary artery endothelial cells HPAEC.  Ref: 14709399 Microvasc Res, 2004
- **Erk**  1 2, Elk 1, and CREB were identified as common targets of PKA,  **PKC** , and CaMK II during memory consolidation the time window and sequence of their phosphorylation was specific for the individual kinase.  Ref: 14739006 Behav Brain Res, 2004
- In 500 microM H 2 O 2 stimulated rat aortic smooth muscle cells, raxofelast dramatically attenuated the activation of mitogen activating protein kinase MAPK  **ERK**  kinase MEK and protein kinase C  **PKC**  without affecting Ras expression.  Ref: 14744595 Eur J Pharmacol, 2004
- The extent duration of phospho  **ERK**  nuclear localization may also be important determinants of the duration of  **PKC**  agonist induced growth arrest in this system.  Ref: 14670956 J Biol ChemJ Biol Chem, 2004
- TGFb1 stimulated glucose transport is not associated with increased expression or membrane incorporation of GLUT, and 10, and depends on  **PKC**  and  **ERK**  activation.  Ref: 14749204 Am J Physiol Endocrinol MetabAm J Physiol Endocrinol Metab, 2004
- Contribution of  **PKC**  dependent and independent processes in the temporal regulation of  **ERK**  by ET 1, EGF and PDGF in rat myometrial cells role in proliferation.  Ref: 14644778 Am J Physiol Cell PhysiolAm J Physiol Cell Physiol, 2004
- Pharmacological analyses using several inhibitors indicated that protein kinase C delta  **PKC**  delta regulates the VEGF induced activation of PLD  **ERK** .  Ref: 14704231 Am J Physiol Heart Circ PhysiolAm J Physiol Heart Circ Physiol, 2004
